# Structural and Functional Characterization of a Ruminal β-Glycosidase Defines a Novel Subfamily of Glycoside Hydrolase Family 3 with Permuted Domain Topology*

**DOI:** 10.1074/jbc.M116.747527

**Published:** 2016-09-27

**Authors:** Mercedes Ramírez-Escudero, Mercedes V. del Pozo, Julia Marín-Navarro, Beatriz González, Peter N. Golyshin, Julio Polaina, Manuel Ferrer, Julia Sanz-Aparicio

**Affiliations:** From the ‡ Department of Crystallography and Structural Biology, Institute of Physical-Chemistry “Rocasolano,” Consejo Superior de Investigaciones Científicas, Serrano 119, 28006 Madrid, Spain,; the § Institute of Catalysis and Petrochemistry, Consejo Superior de Investigaciones Científicas, Marie Curie 2, Cantoblanco, 28049 Madrid, Spain,; the ¶ Institute of Agrochemistry and Food Technology, Consejo Superior de Investigaciones Científicas, Carrer Catedràtic Agustín Escardino Benlloch 7, 46980 Paterna, Valencia, Spain,; the ‖ School of Biological Sciences, Bangor University, LL57 2UW Gwynedd, United Kingdom, and; the ** Immanuel Kant Baltic Federal University, 236040 Kaliningrad, Russia

**Keywords:** enzyme structure, glycoside hydrolase, metagenomics, protein structure, X-ray crystallography, GH3 family, β-glycosidase, enzymology, permuted domains topology, phylogenetic analysis

## Abstract

Metagenomics has opened up a vast pool of genes for putative, yet uncharacterized, enzymes. It widens our knowledge on the enzyme diversity world and discloses new families for which a clear classification is still needed, as is exemplified by glycoside hydrolase family-3 (GH3) proteins. Herein, we describe a GH3 enzyme (GlyA_1_) from resident microbial communities in strained ruminal fluid. The enzyme is a β-glucosidase/β-xylosidase that also shows β-galactosidase, β-fucosidase, α-arabinofuranosidase, and α-arabinopyranosidase activities. Short cello- and xylo-oligosaccharides, sophorose and gentibiose, are among the preferred substrates, with the large polysaccharide lichenan also being hydrolyzed by GlyA_1_. The determination of the crystal structure of the enzyme in combination with deletion and site-directed mutagenesis allowed identification of its unusual domain composition and the active site architecture. Complexes of GlyA_1_ with glucose, galactose, and xylose allowed picturing the catalytic pocket and illustrated the molecular basis of the substrate specificity. A hydrophobic platform defined by residues Trp-711 and Trp-106, located in a highly mobile loop, appears able to allocate differently β-linked bioses. GlyA_1_ includes an additional C-terminal domain previously unobserved in GH3 members, but crystallization of the full-length enzyme was unsuccessful. Therefore, small angle x-ray experiments have been performed to investigate the molecular flexibility and overall putative shape. This study provided evidence that GlyA_1_ defines a new subfamily of GH3 proteins with a novel permuted domain topology. Phylogenetic analysis indicates that this topology is associated with microbes inhabiting the digestive tracts of ruminants and other animals, feeding on chemically diverse plant polymeric materials.

## Introduction

Family 3 of glycoside hydrolases (GH3)^3^ contains about 11,000 entries among which are diverse enzyme activities, including β-glucosidase, β-xylosidase, exo-chitosanase, β-*N*-acetylglucosaminidase, glucocerebrosidase, exo-1,4-β-glucosidase, and exo-1,3/1,4-β-glucanase, that have been characterized (1). A few reported cases are the bifunctional α-l-arabinopyranosidase/β-galactosidase (2), *N*-acetyl-β-glucosaminidase/β-glucosidase (3), β-glucosidase/cellodextrinase (4), β-xylosidase/α-l-arabinofuranosidase (5), and β-glucosidase/β-xylosidase (6). They are retaining enzymes that remove single glycosyl residues from the non-reducing end of their substrates. Therefore, they perform catalysis by a two-step mechanism through a covalent enzyme-glycon intermediate, which is subsequently hydrolyzed via an oxocarbenium ion-like transition state.

Despite the high number of known GH3 sequences, structural knowledge on members of the GH3 family was absent until 1999, when the three-dimensional structure of the β-d-glucan exohydrolase Exo1 from *Hordeum vulgare* (barley) was reported (7). This study showed the core structure of most GH3 enzymes consisting of an N-terminal (α/β)_8_ barrel domain 1, which houses the active site pocket and the nucleophile, and a C-terminal (α/β)_6_-sandwich domain 2, containing the acid/base catalyst. The contribution of different domains in supplying crucial catalytic residues was a highly unusual feature of GH3 enzymes. Furthermore, in the last few years many new structural studies have shown a great variety in domain composition and arrangement of typical GH3 β-glycosidases, having up to four separate domains (8–15). Although this variety produces a shift in the sequence position of the acid/base catalyst, the known structures revealed that its structural location is well conserved among the different members. In contrast, several reported structures have revealed a more uniform pattern of the β-*N*-acetylglucosaminidases (NagZ) members showing that, despite a few having two domains, most Gram-negative bacteria encode single domain enzymes, and all of them have the acid/base catalyst in an unusual histidine/aspartate dyad located in a flexible loop of the (α/β)_8_ barrel (16). This highly mobile loop has been proved to participate in substrate distortion to a ^1^S_3_ conformation, therefore forming a productive Michaelis complex along catalysis (17). This has not been observed in other GH3 enzymes, with the substrate being in a relaxed chair conformation, although a Michaelis complex has been recently reported for the *Listeria innocua* β-glucosidase (18). Among all GH3 β-glycosidases with available structures, insights into the substrate specificity observed in the family has been reported for the *H. vulgare* Exo1 (7, 19–21) or the β-glucosidases from *Thermotoga neapolitana* (8) and *Kluyveromyces marxianus* (9). However, the high varieties in structure and composition found among the different enzymes make it difficult to extrapolate general rules explaining function, and a clear classification of different subfamilies is still needed.

A proper classification of GH3 glycosidases may require extensive biochemical and structural characterization of new enzymes. In this context, nature provides an inexhaustible reservoir from which enzymes can be isolated (22), because they are continuously changing and evolving as a consequence of natural processes of selection. Genomics and metagenomics have made accessible such an enormous reserve of uncharacterized enzymes. Thus, we and others have recently taken advantage of sequencing and extensive screening technologies to develop enzyme discovery strategies and to identify microbial enzymes with improved and unusual activities and specificities (23–25), as well as distinct active site architectures and substrate preferences relative to other structurally characterized enzymes (26). These elegant studies demonstrated that nature contains proteins with novel and/or altered sequences and protein structures, the analysis of which represents one of the major challenges in postgenomic biology (27).

Here, activity screening of a metagenomic library created from rumen fluid led us to the isolation of a novel β-glycosidase, GlyA_1_, which was assigned to the GH3 family. Detailed biochemical characterization of the new enzyme revealed its substrate specificity, whereas its sequence and crystal structure analysis revealed a novel permuted domain topology, defining a new subgroup within the GH3 family. The enzyme contains an additional C-terminal domain, previously unidentified, with its molecular flexibility being explored by small angle x-ray scattering (SAXS) analysis. The structural and biochemical analysis of the GlyA_1_ hydrolase presented in this study shed new light on comparative catalysis and evolutionary model studies as well as phylogenetic relationships.

## Results

### 

#### 

##### Library Screening

A subset of 14,000 clones from resident microbial communities of strained ruminal fluid (SRF) collected from rumen-fistulated, non-lactating Holstein cows (28) was screened for its ability to hydrolyze *p*-nitrophenyl-β-d-glucoside (*p*NPβGlc) and *p*-nitrophenyl-β-d-cellobioside (*p*NPβCel). We identified a positive clone (designated SRF4) that is highly active against both substrates. The fosmid with insert SRF4 (38,710 bp; G + C 41.89%) was fully sequenced. A gene herein designated as *gly*A_1_ encoding a potential GH3 β-glycosidase (GlyA_1_) was identified out of the 38 distinct genes on the hit fosmid. The deduced molecular mass and estimated pI value were 101,849 Da and 4.86, respectively. This 921-amino acid-long putative protein exhibited a maximum amino acid sequence identity of 59% to a similar protein in public databases (with a top hit EDO57841.1 from *Clostridium* sp.). A search of oligonucleotide patterns against the GOHTAM database (29) and TBLASTX analysis revealed compositional similarities between the DNA fragment (38,710 bp) containing the gene for GlyA_1_ with genomic sequences of *Eubacterium, Butyrivibrio,* and *Coprococcus* spp. BLASTN revealed similarities of short DNA fragments to *Prevotella* and *Paenibacillus* spp. BLASTX (search by translated DNA sequences) showed similarity to glycosidases of unknown *Clostridia* (phylum *Firmicutes*). BLASTP search with identified protein sequences showed good matches for many of them against corresponding proteins in *Eubacterium* and *Prevotella* and members of Lachnospiraceae, *Clostridium*, *Ruminococcus,* and *Bacteroides*. Most likely, GlyA_1_ has thus its origin in the phylum Firmicutes, and the presence of a phage gene may, however, indicate a horizontal gene transfer of the carbohydrate metabolism genes from Firmicutes to Bacteroidetes. Those microbes are known to be abundant in the ruminal environment and are thought to play key roles in the breakdown of proteins and carbohydrate polymers (30, 31).

##### Biochemical Characterization of GlyA_1_

**FIGURE 1.**
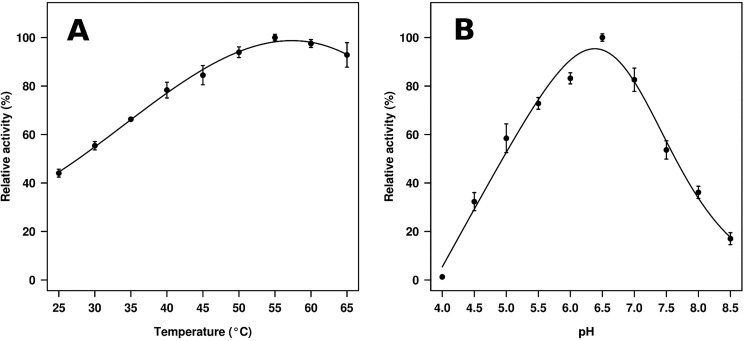
**Temperature (*A*) and pH (*B*) profiles of the purified β-glucosidase GlyA_1_.** The data represent the relative percentages of specific activity (units/g) compared with the maximum activity using *p*NPβGlc as substrate (100% in *A*, 2841 units/g; 100% in *B*, 3056 units/g). The specific activities were calculated using 0.23 μm protein and 10 mg/ml *p*NPβGlc as the assay substrate. *A*, reactions were performed in 50 mm sodium acetate buffer, pH 5.6, at different temperatures. *B*, reactions were performed at different pH (50 mm BR buffer) and at 40 °C. Standard deviations of the results of assays conducted in triplicate are shown.

The gene encoding putative GH3 β-glycosidase (GlyA_1_) was cloned, expressed in *E. coli* BL21 (DE3), and purified. The hydrolytic activity was analyzed using 18 synthetic model *p*-nitrophenyl (*p*NP) derivatives with different sugars as well as a series of 11 additional oligosaccharides. Their specific activities (units/g protein) (Table 1) and the half-saturation (Michaelis) coefficient (*K_m_*), the catalytic rate constant (*k*_cat_), and the catalytic efficiency (*k*_cat_/*K_m_*) values (Table 2) were determined. As shown in Table 1, activity was confirmed for 18 substrates that revealed that GlyA_1_ is a GH3 member with clear β-glucosidase and β-xylosidase activities, but also possessing β-galactosidase, β-fucosidase, α-arabinofuranosidase, and α-arabinopyranosidase activities at low level in this order (Table 1). The activity toward *p*NP-*N*-acetyl-β-d-glucosaminide (*p*NPGlcNAc) and *p*NP-*N*-acetyl-β-d-galactosaminide (*p*NPGalNAc) was below detection limits, and thus the enzyme does not have β-*N-*acetylglucosaminidase nor β-*N*-acetylgalactosaminidase activity. As shown in Table 2, in terms of catalytic efficiencies, *p*NPβCel was the preferred substrate, mainly due to the higher affinity for this substrate as compared with other *p*NP sugars. The purified recombinant hydrolase was also assayed for their activities toward different polymeric substrates. Using specific activity determination, GlyA_1_ hydrolyzed all short cello- and xylo-oligosaccharides tested (degree of polymerization (DP) from 2 to 5), with longer substrates being slightly preferred (Table 1). The catalytic efficiencies (*k*_cat_/*K_m_*) while using the non-activated substrates cellobiose and xylobiose were lower than those found for *p*NPβCel and *p*NP-β-xylobiose (*p*NPβXylb), respectively, mainly due to a significant decrease of *k*_cat_ values for the natural disaccharides (Table 2). A comparison of kinetic parameters using the natural substrates xylobiose and cellobiose and the synthetic *p*NPβXylb and *p*NPβCel substrates confirmed the ∼2-fold higher affinity for oligosaccharides containing β-linked glucosyl *versus* xylosyl substrates. In contrast, the affinities for the monosaccharides *p*NPβGlc and *p*NPβXyl were essentially similar, suggesting that affinity constraints are higher as the size of the oligosaccharides increases. However, due to the differences in *k*_cat_ values, no major differences in catalytic performance were observed when comparing β-Xyl- and β-Glc-containing sugars. The catalytic performance (*k*_cat_/*K_m_*) found for other substrates is from low to very low mainly due to lower catalytic rates. The enzyme also exhibited activity against lichenan, suggesting that is able to hydrolyze substrates with mixed β-1,3/1,4 linkages. No activity was detected using avicel or filter paper, as well as toward substrates without β-1,4 linkages such as β-1,3 glucan or mixed β-1,3/1,6 linkages such as laminarin. Accordingly, the enzyme showed a clear preference for short cello-oligosaccharide substrates, which may likely be produced in natural settings from the cellulose components of plant cell walls due to the action of glucanases in the ruminal fluid. Other substrates such as gentibiose (containing d-glucoses joined by a β-1,6-linkage) and sophorose (or 2-*O*-β-d-glucopyranosyl-α-d-glucose) were also hydrolyzed to a similar extent as cellobiose and xylobiose. The optimum activity for GlyA_1_ was observed within a mesophilic range (45–65 °C) and within a neutral or slightly acid pH (6.0–7.0), being most active at 55 °C and a pH close to 6.5 (Fig. 1).

**TABLE 1:** **Substrate specificity of the purified β-glycosidase GlyA_1_ and truncated GlyA_1_-ΔCt**

Substrate	Specific activity	
	GlyA_1_	GlyA_1_-ΔCt
	*units/g*	
*p*NPβGlc	2226.8 ± 120.2	197.0 ± 10.6
*p*NPβXyl	2876.2 ± 111.1	624.3 ± 34.1
*p*NPβXylb	301.6 ± 8.3	14.8 ± 0.70
*p*NPβCel	290.7 ± 9.9	17.04 ± 0.50
*p*NPβGal	11.8 ± 2.2	0.64 ± 0.01
*p*NPβFuc	1.18 ± 0.01	0.62 ± 0.01
*p*NPαAraf	1.16 ± 0.01	0.64 ± 0.01
*p*NPαArap	0.66 ± 0.01	0.33 ± 0.01
Cellobiose	551.5 ± 2.6	43.6 ± 2.2
Cellotriose	532.6 ± 0.5	44.4 ± 4.9
Cellotetraose	569.3 ± 0.4	48.7 ± 5.1
Cellopentaose	641.5 ± 0.5	53.3 ± 2.1
Xylobiose	634.2 ± 4.3	115.0 ± 4.4
Xylotriose	668.3 ± 7.2	136.1 ± 6.7
Xylotetraose	674.5 ± 11.5	174.7 ± 4.5
Xylopentaose	747.9 ± 3.5	196.4 ± 8.6
Gentibiose	535.2 ± 0.7	45.9 ± 1.2
Sophorose	602.8 ± 0.7	54.3 ± 4.5
Lichenan	68.6 ± 4.8	9.8 ± 0.7

**TABLE 2:** **Kinetic parameters of the purified β-glycosidase GlyA_1_**

Substrate	*K_m_*	*k*_cat_	*k*_cat_/*K_m_*
	*mm*	*s^−1^*	*s^−1^m*^−*1*^
*p*NPβGlc	10.7 ± 2.0	1.63 ± 0.38	152.3
*p*NPβXyl	8.8 ± 0.5	0.95 ± 0.44	107.8
*p*NPβCel	1.4 ± 0.2	0.51 ± 0.15	314.2
*p*NPβXylb	2.5 ± 0.3	0.73 ± 0.12	292.0
*p*NPβGal	7.6 ± 0.1	0.13 ± 0.04	17.1
*p*NPβFuc	4.8 ± 0.3	0.05 ± 0.01	10.4
*p*NPαAraf	7.8 ± 1.7	0.03 ± 0.01	3.85
*p*NPαArap	10.7 ± 3.4	0.01 ± 0.01	0.93
Celobiose	2.4 ± 0.3	0.07 ± 0.01	28.2
Xylobiose	4.7 ± 0.2	0.05 ± 0.01	10.6

##### Biochemical Characterization of GlyA_1_-ΔCt

A mutant containing a missing C-terminal region, herein referred to as GlyA_1_-ΔCt, was created in the vector pQE80L. After purification, activity was determined for the 18 sugars being hydrolyzed by the wild-type enzyme, so the effect of the C-terminal region was tested. As shown in Table 1, the specific activity of the mutant was from 2- to 18.4-fold lower than that of the wild type, suggesting the importance of this region in the overall activity of the enzyme. The negative effect of the elimination of the C-terminal domain (compared with the full-length protein) was most notable for the hydrolysis of sugars containing β-glucose (from 11.3- to 17.1-fold activity reduction) as compared with those containing β-xylose (from 4.6- to 5.5-fold lower activity).

##### Crystal Structure Determination

Preliminary crystals from the wild-type GlyA_1_ were obtained after more than 3 months with PEG3350 as the precipitant, and they were cryoprotected into 25% d-glucose to obtain the complex with this sugar. The structure was solved by molecular replacement using the domains from *T. neapolitana* β-glucosidase as independent search models. Refinement and analysis of electron density maps allowed modeling of the chain containing residues 3–798 but did not show any density to build the C-terminal segment 800–921, suggesting a putative cleavage of this region in the slow crystallization step. The low numbers of crystals impeded analysis of the intact protein by mass spectrometry, but SDS-PAGE analysis of protein solution samples revealed the presence of two bands after incubation at room temperature or treatment with proteases. Therefore, the sample was incubated with subtilisin previously to the crystallization step, which accelerated formation of many good quality crystals, under similar conditions and with the same space group. These crystals were cryoprotected into 20% glycerol, and this molecule was found bound at the active site. Furthermore, crystals from a truncated construct containing residues 2–799 (GlyA_1_-ΔCt) also grew in a week with ammonium sulfate as the precipitant and, despite having different shape, yielded the same cell and space group, which is consistent with the hypothesis that the wild-type sample was cleaved. These crystals were used to obtain the complexes with d-xylose and d-galactose. Many attempts done to crystallize the complete enzyme were unsuccessful. Also, a construct with residues 800–921, containing the isolated C-terminal region (GlyA_1_-Ct), failed to crystallize. Crystallographic data and refinement statistics for the four structures here presented are given in Table 3.

**TABLE 3:** **Crystallographic data of GlyA_1_** Values in parentheses are for the high resolution shell.

Crystal data	GlyA_1_/glycerol	GlyA_1_/glucose	GlyA_1_-ΔCt/xylose	GlyA_1_-ΔCt/galactose
Space group	*P*2_1_2_1_2_1_	*P*2_1_2_1_2_1_	*P*2_1_2_1_2_1_	*P*2_1_2_1_2_1_

**Unit cell parameters**				
*a* (Å)	51.22	50.63	50.60	50.92
*b* (Å)	119.72	119.18	119.32	119.25
*c* (Å)	157.49	157.42	157.20	157.48

**Data collection**				
Beamline	Diamond (IO3)	PetraIII/DESY (P13)	ESRF (ID23–1)	ALBA (XALOC)
Temperature (K)	100	100	100	100
Wavelength (Å)	0.9762	0.9786	0.9762	1.1271
Resolution (Å)	95.31–1.83 (1.83–1.87)	95.03–2.17 (2.17–2.24)	95.05–2.08 (2.08–2.14)	95.08–2.29 (2.29–2.37)

**Data processing**				
Total reflections	537,914 (21,607)	338,356 (28,624)	384,429 (29,703)	287,828 (27,811)
Unique reflections	84,644 (3858)	51,199 (4369)	58,135 (4451)	44,188 (4264)
Multiplicity	6.4 (5.6)	6.6 (6.6)	6.6 (6.7)	6.5 (6.5)
Completeness (%)	98.9 (87.2)	99.7 (99.9)	99.9 (99.9)	100.0 (100.0)
Mean *I*/σ (*I*)	8.7 (2.1)	11.0 (3.3)	10.9 (3.0)	11.4 (3.3)
*R*_merge_*^a^* (%)	13.7 (56.2)	12.7 (57.9)	8.7 (54.0)	9.0 (52.8)
*R*_pim_*^b^* (%)	5.9 (24.9)	5.4 (24.4)	3.6 (22.4)	3.8 (22.3)
Molecules/ASU	1	1	1	1

**Refinement**				
*R*_work_/*R*_free_*^c^* (%)	15.68/17.85	17.48/21.82	17.38/21.25	18.16/22.72

**No. of atoms/average B** (Å^2^)				
Protein	6150/19.55	6121/31.57	6129/41.37	6151/47.72
Carbohydrate	0/0	12/38.94	10/43.17	12/53.10
Water molecules	674/28.82	328/31.23	304/41.10	120/39.00
All atoms	6878/20.66	6466/31.59	6503/41.66	6331/47.88

**Ramachandran plot** (%)				
Favored	98.00	97.00	98.00	98.00
Outliers	0	0	0	0

**r.m.s.d.**				
Bonds (Å)	0.007	0.007	0.008	0.010
Angles (°)	1.209	1.218	1.259	1.417

**PDB codes**	5K6L	5K6M	5K6N	5K6O

*^a^ R*_merge_ = Σ*_hkl_* Σ*_i_*|*I_i_*(*hkl*) − (*I*(*hkl*))|/Σ*_hkl_* Σ*_i_I_i_*(*hkl*), where *I_i_*(*hkl*) is the *i*th measurement of reflection *hkl* and (*I_i_*(*hkl*)) is the weighted mean of all measurements.

*^b^ R*_pim_ = Σ*_hkl_* (1/(*N* − 1)) 1/2 Σ*_i_*|*I_i_*(*hkl*) − *I_i_*(*hkl*)|/Σ*_hkl_* Σ*_i_I_i_*(*hkl*), where *N* is the redundancy for the *hkl* reflection.

*^c^ R*_work_/*R*_free_ = Σ*_hkl_*|*F_o_* − *F_c_*|/Σ*_hkl_*|*F_o_*|, where *F_c_* is the calculated and *F_o_* is the observed structure factor amplitude of reflection *hkl* for the working/free (5%) set.

##### Permuted Domain Topology of GlyA_1_

**FIGURE 2.**
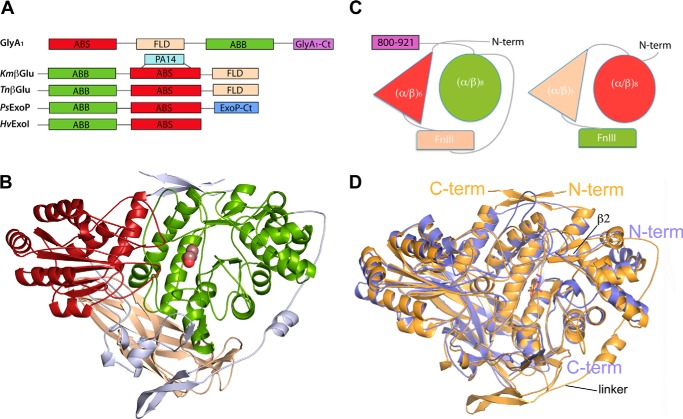
**Permuted domain composition of GlyA_1_.** *A,* comparison of GlyA_1_ structure with representative members of multidomain GH3 enzymes. β-glucosidases from *K. marxianus*, *Km*β*Glu* (9) and *T. neapolitana*, *Tn*β*Glu* (8), the exo-1,3/1,4-β-glucanase from *Pseudoalteromonas* sp., *PsExoP* (10) and the barley β-d-glucan exohydrolase, *HvExoI* (7) are shown. Domains are named as ABS: (α/β)_6_-sandwich; FLD fibronectin-like; ABB (α/β)_8_ barrel; PA14, protective antigen PA14 domain. *B,* folding of GlyA_1_. The N-terminal (α/β)_6_-sandwich domain (*red*) is followed by the FnIII domain (*beige*) and the (α/β)_8_ barrel domain (*green*). Two long segments connect the three domains (*gray*). A glucose found in the active site is represented by *spheres. C,* scheme of the GlyA_1_ domain organization (*left*) as compared with that of *T. neapolitana* β-glucosidase (*right*) (8). *D,* superimposition of GlyA_1_ (*gold*) onto *T. neapolitana* β-glucosidase (*blue*) coordinates. Both enzymes present a deviation from the canonical (α/β)_8_ barrel topology, with their first α-helix missing, which makes strand β2 reversed and antiparallel with the other seven strands. The main difference between both enzymes is the long arm linking the FnIII to the (α/β)_8_ domain in GlyA_1_, which is missing in *T. neapolitana* β-glucosidase. Also, small differences in the orientation of some helixes are observed.

The first solved structure from barley β-d-glucan glucohydrolase (7) showed the core structure common to GH3 enzymes, composed of an N-terminal (α/β)_8_ barrel domain 1 linked to an (α/β)_6_-sandwich domain 2 (Fig. 2*A*); both of them provided residues that make up the active site. The later reported structures from *T. neapolitana* (8), *Trichoderma reesei* (12), *Aspergillus* (13, 14), and *L. innocua* (18) β-glucosidases, and a β-glucosidase isolated from soil compost (32), showed the presence of an additional fibronectin type III (FnIII) domain (also designated fibronectin-like domain or FLD) located at the C terminus. This three-domain arrangement is shared by other reported β-glucosidases from *K. marxianus* (9) and *Streptomyces venezuelae* (11) that also contain an additional PA14 domain inserted within the same loop of their (α/β)_6_-sandwich, although both are arranged in a different orientation. Moreover, the structure of the *Pseudoalteromonas* sp. exo-1,3/1,4-β-glucanase has been reported to have a C-terminal domain attached to the core structure, structurally related to family 30 carbohydrate-binding modules (CBM30), although its function is unknown (10). To expand even more this diverse landscape, GlyA_1_ presents a novel structural arrangement showing permuted sequence and topology, in which the (α/β)_6_ sandwich (previous domain 2) is located at the N terminus and the FnIII domain is sequentially inserted between this and the (α/β)_8_ barrel (Fig. 2*A*). Additionally, a 120-residue segment attached to the C terminus most surely folded into an additional domain.

Fig. 2*B* displays the 3D structure of the solved 3–798 region of GlyA_1_, which present overall dimensions of 85 × 65 × 45 Å. The N-terminal (α/β)_6_-sandwich domain (*red,* residues 10–219) is followed by the FnIII domain (*beige,* residues 278–419) and the (α/β)_8_ barrel domain (*green,* residues 468–780). Two long segments connect the three domains (Fig. 2*B, gray*). Linker 1 (residues 220–277) and half of linker 2 (residues 411–443) are tightly wrapped over the core structure, whereas the rest of linker 2 (444–467) forms an extended arm that clasps the (α/β)_8_ barrel. Finally, the regions at the beginning and the end of the chain are making a two-stranded β-sheet that laces the core structure at the top.

Comparative analysis using the Dali (33) server revealed that the GlyA_1_ (α/β)_6_-sandwich domain, containing the catalytic acid/base residue Glu-143, superimposes onto the corresponding domain from the *T. neapolitana* β-glucosidase, with a root-mean-square deviation (r.m.s.d.) of 1.6 Å for 202 eq Cα positions (39% sequence identity). The same comparison with the other structurally known GH3 gives deviations in the range 2–2.5 Å (20–25% sequence identity). The FnIII domain seems more structurally conserved along the GH3 family; GlyA_1_ is most similar to those in the β-glucosidases from *T. neapolitana*, with r.m.s.d. = 1.5 Å (122 residues, 39% identity), and *K. marxianus*, with r.m.s.d. = 1.6 Å (123 residues, 32% identity), but the same analysis gives values in the range 1.8–1.9 Å (21–28% sequence identity) against the other GH3 enzymes containing this domain. Finally, the (α/β)_8_ barrel, which contains the nucleophile Asp-709, is most similar to the corresponding domain in the β-glucosidases from *T. neapolitana* (r.m.s.d. = 1.6 Å, 285 residues, 39% identity), *K. marxianus*, (r.m.s.d. = 1.5 Å, 276 residues, 34% identity), *S. venezuelae* (r.m.s.d. = 1.7 Å, 278 residues, 32% identity), and *T. reesei* (r.m.s.d. = 2.0 Å, 278 residues, 27% identity). Equally to GlyA_1_, all these domains present a deviation from the canonical (α/β)_8_ barrel topology, which was first observed in the *T. neapolitana* β-glucosidase. Thus, their first α-helix of the eight β-α motifs is missing, which has the consequence of making strand β2 reversed and antiparallel with the other seven strands. The different deviation from the canonical topology found at this domain is consistent with the higher deviations found in the structural comparison of GlyA_1_ with other GH3 enzymes, in the range 2.5–3 Å (16–20% identity).

Interestingly, the GlyA_1_ core is structurally rather conserved with known β-glucosidases with equivalent domain architecture (Fig. 2*C*). The superposition of the *T. neapolitana* β-glucosidase onto the structure of GlyA_1_ reported here shows small differences in the orientation of some of the helices (Fig. 2*D*). The main difference is the long arm that links the FnIII to the (α/β)_8_ domain in GlyA_1_, which is missing in *T. neapolitana* β-glucosidase. There are also significant differences in the loops surrounding the active site both in length and orientation, which must be related to the different substrate specificity, as commented below.

##### Architecture of the Active Site

**FIGURE 3.**
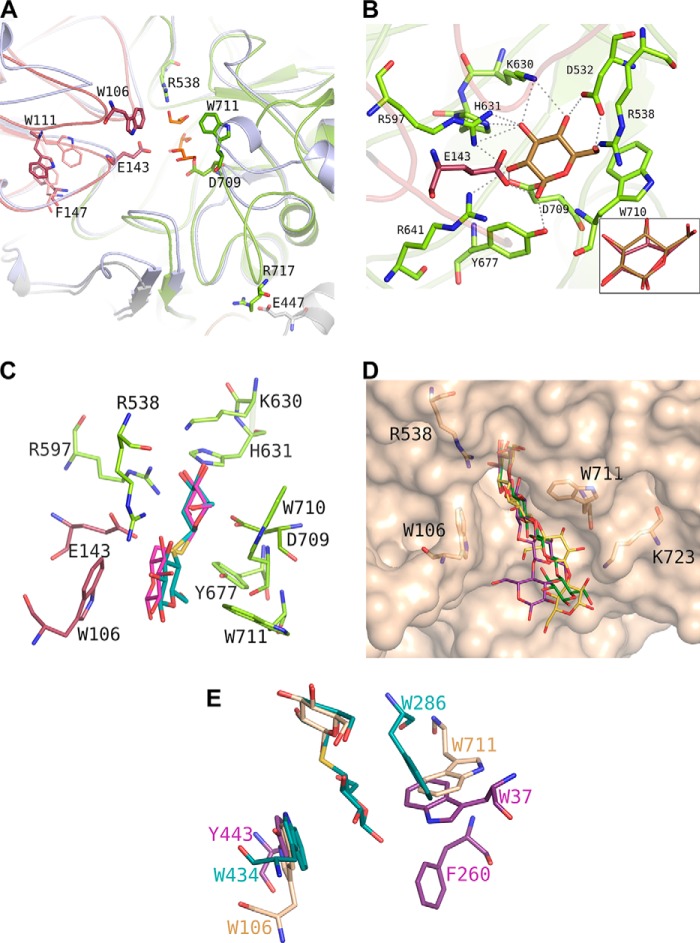
**GlyA_1_ active site architecture.** *A,* detail of the loops surrounding its active site from the (α/β)_8_ barrel (*green*) and the (α/β)_6_-sandwich (*raspberry*) domains, superimposed onto the *T. neapolitana* β-glucosidase (8) (*pale blue*). Three glycerol molecules from the cryobuffer found in the GlyA_1_ crystals are shown in *orange*. Asp-709 and Glu-143 are the nucleophile and the acid/base catalyst, respectively. Main features of GlyA_1_ are the extended loop containing Asp-709, which includes Trp-711 and the ion pair Arg-717–Glu-447 fixing it to the unique long arm and a highly flexible loop containing Trp-106. Two different conformations found among the crystals at Trp-111 and Phe-147 are highlighted. *B,* detail of the atomic interactions defining subsite −1. A glucose molecule is shown in *gold*. Xylose binds in the same relaxed chair conformation, and only interaction of the glucose O6 hydroxyl is missing. *Inset,* binding mode of galactose in a semi-chair conformation by flattening of the C4 atom that has the axial hydroxyl substituent and keeping the same interaction pattern. *C,* thiocellobiose (*cyan*) and thiogentibiose (*pink*) modeled at the active site by structural superimposition to the previously determined β-d-glucan glucohydrolase barley complexes (PDB entries 1IEX and 3WLP (34)), delineating putative subsite +1. *D,* molecular surface of the GlyA_1_ active site, with relevant residues as *sticks.* Three different β-1,4/β-1,3-linked tetraglucosides have been manually docked by superposition of their non-reduced end to the experimental glucose: a cellotetraose, as found in PDB entry 2Z1S (*green*); a Glc-4Glc-3Glc-4Glc (*purple*), and a Glc-4Glc-4Glc-3Glc (*yellow*), as built by the on-line carbohydrate-building program GLYCAM (45) and exported in its minimum energy state. *E,* superposition of GlyA_1_-Glc structure (*beige*) with those reported for *T. reesei* β-glucosidase (*purple*) (12) and barley β-d-glucan glucohydrolase complexed with thiocellobiose (*cyan*) (34).

The active site of GlyA_1_ is located at the molecular surface, at the interface between the (α/β)_8_ barrel domain, which provides the nucleophile Asp-709 and the (α/β)_6_-sandwich domain, contributing to the Glu-143 acid/base catalyst (Fig. 3*A*). The participation of Asp-709 in substrate hydrolysis was confirmed by site-directed mutagenesis (D709A) in GlyA_1_ and GlyA_1_-ΔCt, as *K_m_* and *k*_cat_ values could not be determined from the data obtained due to the activity value being below the detection limit. It is a pocket of 12 Å deep with a narrow entrance 4–6 Å wide. A detailed structural comparison with the *T. neapolitana* β-glucosidase (Fig. 3*A*) reveals the main differences in loop conformation observed around the active site that are responsible for making a deeper catalytic pocket in GlyA_1_. First, loop β7-α7 of the (α/β)_8_ barrel, following the nucleophile Asp-709 (residues 711–726), has an 11-residue insertion that extends away from the pocket and interacts with the long segment linking the FnIII domain to the barrel, which is missing in the *T. neapolitana* β-glucosidase. Here, Arg-717 makes an ion pair with Glu-447 at the small helix located in the middle of the extended linker, which helps in stabilizing this region. An important feature of this β7-α7 loop is the presence of Trp-711, close to the nucleophile Asp-709, that protrudes from the surface and delineates a narrow catalytic pocket. Moreover, and despite loop β3-α3 (residues 536–550) being shorter in GlyA_1_, Arg-538 clearly bulges into the pocket contributing to constrict it even more.

With respect to the (α/β)_6_-sandwich, similarly to that observed in *T. neapolitana* β-glucosidase, this domain is shaping the active site by means of two loops, residues 139–152 containing the acid/base catalyst Glu-143 and residues 100–113 enclosing Trp-106 that clearly projects into the catalytic pocket. Interestingly, the last loop is markedly flexible as it is deduced from the fact that it could only be fully traced in the ligand-free crystal, containing only glycerol in the active site, and in the galactose-soaked crystal of the truncated form. In contrast, the crystals of the full-length and truncated forms, soaked into glucose and xylose, respectively, showed poor density that precluded tracing residues 104–107. Furthermore, the traced loops showed significant conformational changes in the different crystals at Trp-111, coupled to a change in Phe-147 from the adjacent 139–152 loop (Fig. 3*A*), reinforcing its intrinsic mobility. The loop equivalent to 100–113, which is highly variable within GH3 enzymes, was proposed to be involved in recognition of large substrates from the crystal structure of *T. neapolitana* β-glucosidase, which showed some disorder that precluded tracing of a segment equivalent to that not observed in some GlyA_1_ crystals. Noteworthy, the non-visible region of *T. neapolitana* β-glucosidase includes Trp-420 that, consequently, may be defining additional binding subsites, similarly to Trp-106. However, the remaining sequence is not conserved, with both Phe-147 and Trp-111 being unique to GlyA_1_, and therefore, the substrate recognition mode presented by the two enzymes to accommodate the substrate may be different.

Soaking with xylose and glucose showed a clear density indicating that both sugars occupy the catalytic pocket subsite −1 in a relaxed chair conformation (Fig. 3*B*). This subsite is well conserved among known GH3 β-glucosidases and, with the exception of the acid base catalyst, is made up entirely by residues from the (α/β)_8_ barrel domain. Thus, residues from the loops emerging from the central β-strands are making a tight net of hydrogen bonds that accommodate the glycon with all its OH groups making at least two polar interactions. The glycon moiety is located by stacking to Trp-710, and the acid base catalyst Glu-143 and the nucleophile Asp-709 interact with the O1 and O2 hydroxyls, as is expected in GH enzymes. The other residues making subsite −1 are Asp-532, Arg-597, Lys-630, His-631, Arg-641, and Tyr-677. Xylose and glucose are bound in an identical position, and the glycerol molecules observed in the ligand-free crystals are mimicking the positions occupied by C2, C3, C4, and C5 from both sugars. The additional polar interaction made by the glucose O6 hydroxyl appears consistent with the higher affinity observed in GlyA_1_ toward glucosides as compared with xylosides. Thus, as shown in Table 2, the affinity for cellobiose (*K_m_* = 2.4 ± 0.3 mm) was ∼2-fold higher than that for xylobiose (*K_m_* = 4.7 ± 0.2 mm). Interestingly, soaking of crystals with galactose showed that this sugar displays a semi-chair conformation at subsite −1 by flattening of the C4 atom that has the axial hydroxyl substituent (Fig. 3*B*, *inset*). In this way, galactose is accommodated by essentially the same polar interactions observed in the glucose complex, thereby explaining the activity of the enzyme on β-galactosides. However, the energy cost of getting the substrate ring distortion is reflected by the lower β-galactosidase activity, as given in Tables 1 and 2. Accordingly, the low β-fucosidase and α-arabinosidase activities must reflect some degree of deviation from the glucose-binding pattern, through ring distortion and/or loss of polar interactions, but in any case the plasticity of the catalytic site provides a notable capacity of GlyA_1_ to accept different sugars (from high to low and very low specificity)_._

As said before, and in contrast to that observed in *T. neapolitana* β-glucosidase that presents an active site opened to the solvent with only subsite −1 being defined, more subsites are apparent in GlyA_1_. To delineate a putative +1 subsite, we modeled the position of the non-hydrolysable substrate analogs thiocellobiose and thiogentibiose by structural superimposition on the previously reported experimental barley complexes (34). As shown in Fig. 3*C*, Trp-106 and Trp-711 define a hydrophobic patch that may allocate the oligosaccharides at a putative subsite +1, leaving a range of possible ring orientations compatible with the observed activity of GlyA_1_ against differently β-linked bioses, as given in Table 1. Also, the long chain of Arg-538, protruding at the catalytic pocket as said above, is in good position to stabilize the sugar unit by making hydrogen bonds to one or possibly two of its hydroxyl groups. The important contribution of subsite +1 to GlyA_1_ substrate binding efficiency (both glucosides and xylosides) is manifested by the lower *K_m_* value with *p*NPβCel compared with *p*NPβGlc and by the lower *K_m_* value with *p*NPβXylb compared with *p*NPβXyl (Table 2).

Furthermore, inspection of the molecular surface of the active site cavity shown in Fig. 3*D* suggests the possible existence of additional subsites, which is illustrated by several β-1,4/1,3-linked oligosaccharides that have been modeled at the active site as follows: a glucotetraose (*green*), a Glc-4Glc-3Glc-4Glc chain (*purple*), and a Glc-4Glc-4Glc-3Glc (*yellow*). These sugars have been docked by superimposition of their non-reducing units onto the observed glucose at the GlyA_1_ complex. The hydrophobic patch defined by Trp-106 and Trp-711 may fit the oligosaccharides at subsites +1 and +2, and the long side chain of Lys-723 seems available to make polar interactions with the hydroxyl groups defining a possible subsite +3. The putative existence of at least three subsites in the GlyA_1_ active site would be in agreement with the tendency of an increased activity against longer cello- and xylo-oligosaccharides (see Table 1). Also, the tendency of increased activity against longer cello- and xylo-oligosaccharides as given in Table 1 suggests interactions at more distal positions and therefore the possibility of additional subsites. Moreover, the shape of the active site seems compatible with the mixed β-1,4/1,3-links of the modeled tetrasaccharides, thereby explaining the observed activity on the medium size polymer lichenan.

Comparison of the GlyA_1_-Glc structure with those reported for *T. reesei* β-glucosidase (12) and barley β-d-glucan glucohydrolase complexed with thiocellobiose (Fig. 3*E*) (7) displays the different hydrophobic platforms found at each active site. The barley β-d-glucan glucohydrolase structure showed a narrow channel with the glucose tightly arranged at subsite +1, being sandwiched between Trp-286 and Trp-434 side chains. In contrast, the GlyA_1_ Trp-711 is perpendicular and oriented similarly to Trp-37 found in *T. reesei* β-glucosidase, although both residues are provided by different loops from the (α/β)_8_ barrel domain. At the opposite face, GlyA1 Trp-106 is structurally equivalent to Tyr-443 and Trp-434 from the barley and *T. reesei* enzymes, although all of them come from different loops within the (α/β)_6_-sandwich domain. Interestingly, other enzymes present an aromatic residue in a position identical to Trp-106, but they are provided by the PA14 domain, Phe-508 in the case of the *K. marxianus* β-glucosidase, or by a long loop coming from the other subunit, Tyr-583 in the case of the *L. innocua* β-glucosidase dimer (data not shown) (18). This feature illustrates that these highly diverse enzymes have evolved common topology and molecular mechanisms, and yet the precise structural differences behind that regulate specificity.

##### SAXS Analysis of GlyA_1_

Because of the unfeasibility in crystallizing the full-length GlyA_1_, we explored its overall flexibility and putative shape in solution by SAXS experiments. Thus, we compared the molecular descriptors of the complete construct with respect to the truncated construct GlyA_1_-ΔCt, lacking the C-terminal domain. For this purpose, several solutions with varying concentrations were measured for each sample, and their scattering curves were merged to extrapolate idealized data. Analysis of the scattering curves shows a good fit to the Guinier approximation, which indicates that the samples are not aggregated. Also, the calculated radii of gyration (*R_g_*) are consistent across the range of measured concentrations. Then, the overall size descriptors can be properly determined for each construct.

First of all, the calculated molecular masses from both samples are close to the expected values (Table 4), indicating the presence of monomers, and also a 15-kDa higher mass in the complete protein, which excludes proteolysis of the analyzed sample in the short time of the experiment. Furthermore, the *R_g_* and the maximum distance (*D*_max_) for the complete protein are only slightly higher than the truncated protein, which may be indicate that the extra C-terminal domain is not too extended from the core structure. In support of this hypothesis, the pairwise distance distribution function *P*(*r*) calculated for both constructs shows a similar unimodal pattern consistent with a single domain protein in both cases. Furthermore, the analysis of the scattering function by the Kratki plots is consistent with the expected profile for a folded protein with a clear peak, in contrast what is observed in multidomain proteins with flexible linkers that present several peaks or smoother profiles. Consequently, we do not observe in the data calculated from the complete protein any of the signs that may be indicative of molecular flexibility, *i.e.* large *R_g_* and *D*_max_, absence of correlation in the *P*(*r*) function, or smooth Kratky plots. Therefore, SAXS analysis appears consistent with a compact overall shape of the complete GlyA_1_, in which the extra C-terminal region would not define a marked separate or flexible domain but rather it could be folded over the core three-domain structure.

**TABLE 4:** **SAXS data collection and derived parameters**

Protein	Merged data	From Guinier			From Gnom			From Porod	
		*R_g_*	Quality	*I*(0)	*R_g_*	*I*(0)	*D*_max_	Porod volumen	Mass
	mg/ml	nm	%		nm		nm	nm*^3^*	kDa
GlyA_1_-ΔCt	0.34–5.29	2.784	88	76.38	2.80	76.04	8.479	122.71	72.18
GlyA_1_	0.32–5.04	2.918	84	83.41	2.95	83.92	9.120	148.76	87.51

**FIGURE 4.**
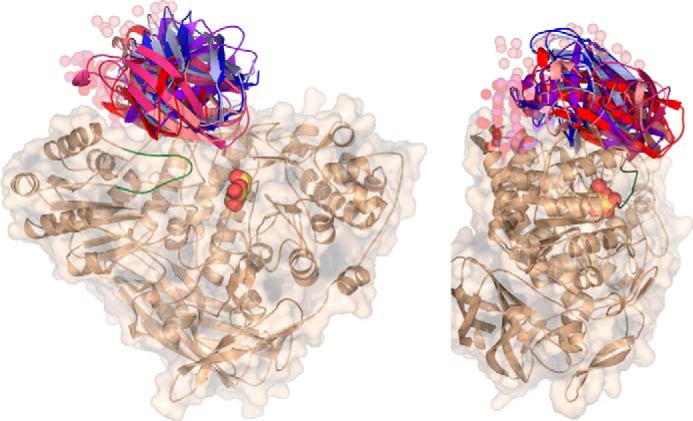
**SAXS analysis of GlyA_1_.** Six *ab initio* models were generated for complete GlyA_1_ from SAXS data, using the experimental structure of the truncated protein and two different models of the last 120 residues (GlyA_1_-Ct). The two templates were obtained from Swiss-Model (*red*) (48) or CPHmodel (*blue*) (49) servers, which predict different lengths of the linker attaching this domain to the core protein, 32 or 5 residues, respectively. CORAL (47) modeling of this linker in each run is represented in *spheres*. The active site pocket is indicated by the galactose found at the crystal (*yellow*), and the mobile loop (residues 100–113), as observed in the galactose-soaked crystals, is highlighted in *green*.

To test the feasibility of this hypothesis, *ab initio* models were generated for complete GlyA_1_ from SAXS data. First, two models of the last 120 residues (GlyA_1_-Ct) were obtained, as explained under “Experimental Procedures,” with both showing an overall β-sandwich topology. This topology is related to carbohydrate-binding domains within families CBM6 and CBM35, to which GlyA_1_-Ct presents 15–20% sequence identity, although the equivalent carbohydrate-binding motifs, typically clusters of conserved aromatic residues, are not evident in its surface. Then, three runs of CORAL were computed by considering the experimental structure of the truncated protein and each of the two models. The six models obtained are shown in Fig. 4. Analysis of these models reveals that all of them cluster around a reduced area that would locate the C-terminal region relatively distant from the catalytic pocket but quite near the mobile loop (residues 100–113). Overall, these models are consistent with the hypothesis proposed above, suggesting that GlyA_1_-Ct may be somewhat packed between the two domains making the core structure and, interestingly, with a putative linker somehow exposed to solvent. This feature might possibly explain the proteolysis observed in the complete protein.

##### GlyA_1_ Phylogenetic Analysis

**FIGURE 5.**
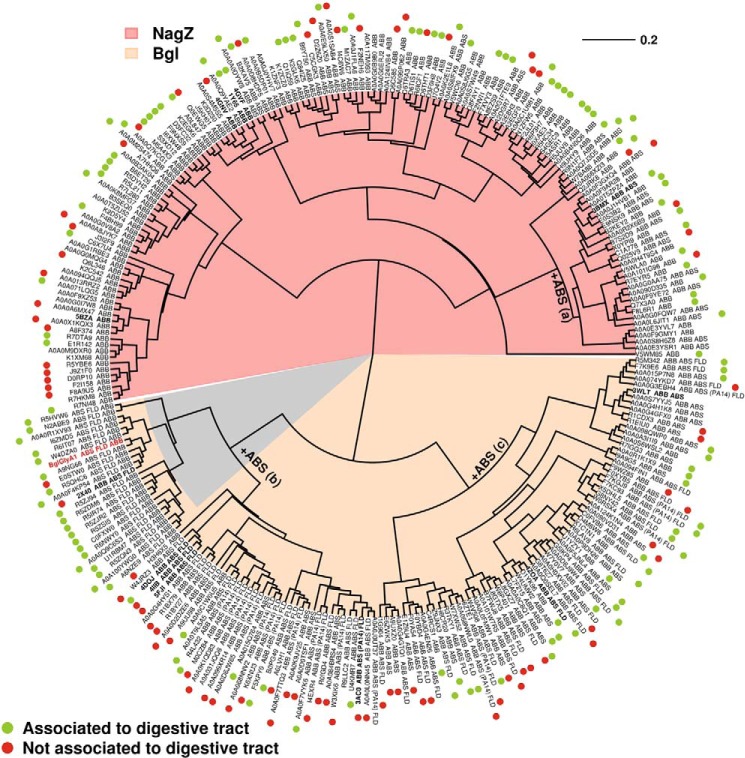
**GlyA_1_ phylogenetic analysis.** The unrooted circular Neighbor-Joining tree indicating phylogenetic positions of polypeptide sequences of the GlyA_1_ enzyme characterized in present work (*red boldface*) and reference similar enzymes. GenBank^TM^ or PDB (in *boldface*) accession numbers are indicated. The domain architecture (ABB, ABB_ABS, ABB_ABS_FLD, ABB-ABS(PA14)-FLD, and ABS_FLD_ABB) to which each sequence is associated is specifically indicated. Multiple protein alignment was performed using ClustalW program, built into software version 2.1. Phylogenetic analysis was conducted with the Ape package implemented for R programming language. Sequences resembling NagZ (β-*N*-acetyl-glucosaminidase) are highlighted with *pink background*. Those encoding GH3 β-glucosidases are indicated in *brown*; within them, those with GlyA_1_-like permuted domain topology are indicated in *gray. ABB,* (α/β)_8_ barrel; *ABS* (α/β)_6_-sandwich; *FLD*, fibronectin-like type III domain; *PA14,* protective antigen PA14 domain.

Our structural analysis illustrated that the permuted domain architecture of GlyA_1_ keeps the location of the active site at the interface between the (α/β)_8_ barrel and the (α/β)_6_-sandwich domains. As mentioned above, *N*-acetylglucosaminidases are built by a single domain, with its (α/β)_8_ barrel holding both the nucleophile and acid/base catalyst. Interestingly, the *Bacillus subtilis* NagZ shows the two-domain composition but still keeps the catalytic residues at the (α/β)_8_ barrel (16). Therefore, this domain may be considered as the characteristic signature of GH3 enzymes. To examine the phylogenetic positioning of β-glucosidases with inverted topology (represented by GlyA_1_) within the GH3 family, we have carried out a phylogenetic analysis based on the sequence of its (α/β)_8_ barrel domain (ABB in this analysis). Sequences representative for each of the domain architectures found in the GH3 domain were selected (details under “Experimental Procedures”). The five topologies selected for this study are ABB, ABB-ABS, ABB-ABS-FLD, ABS-FLD-ABB, and ABB-ABS(PA14)-FLD (ABS (α/β)_6_-sandwich; FLD is fibronectin-like type III domain). The resulting phylogenetic tree given in Fig. 5 shows apparent correlation between ABB sequence divergence and domain architecture. Most single domain sequences (ABB) cluster together and correspond to *N*-acetylglucosaminidases (Fig. 5, *salmon area* of the tree). Insertion of the ABS module is associated with three different nodes (*a, b,* and *c* in Fig. 5). Insertion at node *a* was not accompanied by a significant divergence in the ABB sequence because both ABB and ABB-ABS architectures appear mixed at this node. In fact, these ABB-ABS sequences also correspond to *N*-acetylglucosaminidases, and crystallographic data of *B. subtilis* NagZ show that the two modules are quite independent from a structural point of view. ABS insertion at nodes *b* and *c* would correspond to the divergence of GH3 enzymes giving rise to other activities, mainly β-glucosidase. Within node *c*, other modules (FLD and PA14) were appended after ABS. At node *b*, fusion of C-terminal FLD seems to occur close to ABS addition because most sequences contain both modules. GlyA_1_ and the other GH3 enzymes with inverted topology arose within this cluster. The phylogenetic analysis shows that the inverted topology is predominantly found in Firmicutes, although it is also present in at least another phylum (Actinobacteria) and even Archaea. Furthermore, it appears clearly associated to enzymes belonging to bacteria dwelling in the digestive tract of animals.

## Discussion

In this work, a functional metagenome library analysis was used to identify a β-glycosidase from a plant polymer-degrading microorganism populating the rumen of a dairy cow. The enzyme most likely originated from the genome of a representative of Firmicutes phylum known to be abundant in the ruminal environment (30, 31).

The structural and biochemical analysis of the GlyA_1_ hydrolase presented in this study sheds new light on the mechanisms of the catalysis and evolutionary patterns of the GH3 family. Our data demonstrated that GlyA_1_ has a permuted domain topology. It is well documented that the formation of new domain combinations is an important mechanism in protein evolution. The major molecular mechanism that leads to multidomain proteins and novel combinations is non-homologous recombination, sometimes referred to as “domain shuffling.” This may cause recombination of domains to form different domain architectures. Proteins with the same series of domains or domain architecture are related by descent (*i.e.* evolved from one common ancestor) and tend to have the same function (35), which is rarely the case if domain order is switched. Indeed, a detailed analysis of the structures of proteins containing Rossmann fold domains demonstrated that the N- to C-terminal order of the domains is conserved because the proteins have descended from a common ancestor. For pairs of proteins in the PDB in which the order is reversed, the interface and functional relationships of the domains are altered (36). This was also proved in this study, which revealed that the altered domain architecture in GH3 mostly evolved from a distinct ecological niche, most likely from digestive tracts, including that of the ruminants. Also, the substrate specificity of the GlyA_1_ protein is markedly different from that of reported GH3 members. Indeed, GlyA_1_ is a uncommon multifunctional GH3 with β-glucosidase, β-xylosidase, β-galactosidase, β-fucosidase, α-arabinofuranosidase, α-arabinopyranosidase, and lichenase co-activities, with the ability to degrade β-1,2-, β-1,3-, β-1,4-, and β-1,6-glucobioses.

From an ecological point of view, the rumen compartment provides stable and favorable conditions for microbial growth and is also permanently exposed to plant biomass; for this reason, it contains specialized microorganisms that are permanently competing or collaborating for the degradation of the plant fibers. The data herein suggest that this factor, namely the high exposure to plant biomass, which is less common in other habitats, may be a strong force driving the establishment of gut microbiota with GH3 protein with permuted structures that may provide ecological advantages. Indeed, the permuted domain topology may confer the protein different functionalities such as the ability to expand the pool of biomass-like substrates being hydrolyzed. Overall, our results (analysis of oligonucleotide pattern and phylogenetic tree) strongly suggest that GlyA_1_ and related GH3 enzymes with inverted topology emerged in Firmicutes, where their presence is rather frequent, and are transferred by horizontal gene transfer to bacteria from other phyla and even to another kingdom (Archaea). It is well documented that these wide ranging gene transfer events take place at high frequency in the rumen (37, 38). Probably, GlyA_1_ topology arose from a sequence encoding a GH3 enzyme with ABB-ABS-FLD domain architecture by gene inversion. Although the inversion surely rendered a nonfunctional gene, further mutations that would restore some sort of glycolytic activity would be strongly favored by selective pressure.

Structural analysis illustrates the permuted domain composition of GlyA_1_ that is composed of an N-terminal (α/β)_6_-sandwich domain, followed by the FnIII domain, and the (α/β)_8_ barrel domain. Based on sequence data, a C-terminal domain was expected after the (α/β)_8_ barrel domain. However, attempts to crystallize the C-terminal region of the protein were unsuccessful, and its functional role was unclear. Biochemical characterization of the GlyA_1_ and GlyA_1_-ΔCt proteins revealed that the C-terminal domain does not affect the overall substrate profile of the protein, but rather it affects the catalytic performance, which is significantly lower in the truncated GlyA_1_-ΔCt protein. This suggests that most likely the C-terminal domain may not have a direct role in substrate binding, but still it might disturb the dynamics of the proximate mobile loop (residues 100–113), which seems directly involved in catalysis.

According to available structure-prediction tools, this C-terminal region is expected to adopt a lectin-like topology, related to the CBM6/CBM35 domains. However, it does not seem an obvious carbohydrate-binding domain, and in fact, binding to xylan, cellulose, and barley glucan was not observed by affinity gel electrophoresis assays (data not shown). Nevertheless, although its involvement in binding small substrates does not seem apparent, this domain might be playing a role in positioning or locating the enzyme to distal positions of a yet unknown polymeric substrate by recognizing specific but still unidentified substitutions. Alternatively, it could play a role in keeping the enzyme attached to the cell surface, facilitating the intake of its products and conferring the bacteria an advantage over competing organisms. Interestingly, the analysis of the GlyA_1_-Ct homologous sequences shows that these domains are attached to GH3 β-glucosidases from a ruminal environment, and this feature points to a possible function related to this ecosystem. However, its presence is not related to the permuted domain topology, as only half of the sequences included in the GlyA_1_ cluster (Fig. 5) contain segments equivalent to GlyA_1_-Ct.

In conclusion, the analysis of GlyA_1_ here presented uncovers new features of GH3 enzymes and provides a template for a novel subfamily, including members with permuted domain topology. It also allows picturing the GlyA_1_ active site architecture and the molecular basis of its substrate specificity. More work is needed to have a complete picture of the intricate molecular mechanisms that these highly diverse enzymes have evolved to tailor specificity. It will contribute to improve our knowledge about enzymatic carbohydrate degradation and open up new avenues for biocatalysis.

## Experimental Procedures

### 

#### 

##### Reagents and Strains

Chemicals and biochemicals were purchased from Sigma and Megazyme (Bray, Ireland) and were of pro-analysis (p.a.) quality. The oligonucleotides used for DNA amplification were synthesized by Sigma Genosys Ltd. (Pampisford, Cambs, UK). The *E. coli* Rosetta2 (Novagen, Darmstadt, Germany) for cloning and expression of wild-type protein and the genetic constructs in pQE80L vector were cultured and maintained according to the recommendations of the suppliers.

##### Metagenomic Library Screening and Positive-insert Sequencing

A pCC1FOS fosmid metagenomic library created from microbial communities from SRF of rumen-fistulated non-lactating Holstein cows was used. The construction and characteristics of the library were described previously (28). A subset of 14,000 clones were plated onto large (22.5 × 22.5 cm) Petri plates with Luria Bertani (LB) agar containing chloramphenicol (12.5 μg/ml) and an arabinose-containing induction solution (Epicenter Biotechnologies) at a concentration (0.01% w/v) recommended by the supplier to induce a high fosmid copy number. After overnight incubation at 37 °C, the clones were screened for the ability to hydrolyze *p*NPβGlc and *p*NPβCel. For screens, the plates (22.5 × 22.5 cm; each containing 2,304 clones) were covered with an agar-buffered substrate solution (40 ml of 50 mm sodium acetate buffer, pH 5.6, 0.4% w/v agar and 5 mg/ml of *p*NPβGlc and *p*NPβCel as substrates). Positive clones were detected by the formation of a yellow color. One positive clone, herein designated as SRF4, was selected, and its DNA insert was fully sequenced with a Roche 454 GS FLX Ti sequencer (454 Life Sciences, Branford, CT) at Life Sequencing S.L. (Valencia, Spain), and the predicted genes were identified as described previously (28).

##### Cloning of glyA_1_ and Genetic Constructs in pQE80L Plasmid

The full coding sequence of GlyA_1_ (residues 2–921) and a deleted version (residues 2–799) lacking the C-terminal domain (GlyA_1_-ΔCt) were amplified by PCR with 4GF (CACGAGCTCAATATTGAAAAAGTGATACTTGATTGG) as forward oligonucleotide and 4GR1 (AGCCGTCGACTTACTGCTGCTTTTTAAACTCTATTCG) or 4GR2 (AGCCGTCGACTTACACTCTTCCTGCTATCTCAACC) as reverse oligonucleotides, respectively. The SRF4 fosmid was used as the template. The PCR conditions were as follows: 95 °C for 120 s, followed by 30 cycles of 95 °C for 30 s, 55 °C for 45 s, and 72 °C for 120 s, with a final annealing at 72 °C for 500 s. The PCR products were analyzed and agarose gel-purified using the Mini Elute gel purification kit (Qiagen, Hilden, Germany). The PCR products were digested with SacI/SalI and cloned in vector pQE80L to generate plasmids GlyA_1_-pQE and GlyA_1_ΔCt-pQE, respectively. The coding sequence of the C-terminal domain (GlyA_1_-Ct, residues 800–921) was amplified with oligonucleotides CT1F (CACGAGCTCATAGAAGAGGATGCATTCGATATAG) and 4GR1 and cloned in the SacI/SalI sites of pQE80L (plasmids Ct-pQE). GlyA_1_-pQE was used as a template to introduce the mutation D709A by PCR with primers M1 (TGGTGGGCTCAGGTTAATGACC) and M2 (G**G**CAGTCATCACAATACCCTTAAAGCC), as described previously (39). The coding region of the resulting plasmids was fully sequenced to check for the absence of undesired mutation. The *E. coli* strain Rosetta2 (Novagen, Darmstadt, Germany) was transformed with the selected plasmids; the clones were selected on LB agar supplemented with ampicillin (100 μg/ml) and chloramphenicol (68 μg/ml) and stored with 20% (v/v) glycerol at −80 °C.

##### Site-directed Mutagenesis

Mutation D709A was introduced into the corresponding pQE80L plasmids containing genes encoding GlyA_1_ and GlyA_1_-ΔCt, using the QuikChange II XL mutagenesis kit from Agilent Technologies, Inc. (Santa Clara, CA), with TGGTGGGCTCAGGTTAATGACC and GGCAGTCATCACAATACCCTTAAAGCC as forward and reverse oligonucleotides, respectively. The resulting variant plasmids were transferred into *E. coli* strain Rosetta2 (Novagen, Darmstadt, Germany) and selected on the LB agar supplemented with the same antibiotics as parental plasmids.

##### Gene Expression and Protein Purification

For enzyme expression and purification of wild-type and mutant GlyA_1_ and GlyA_1_-ΔCt variants, as well as GlyA_1_-Ct in the pQE80L vector, a single colony (*E. coli* Rosetta2) was grown overnight at 37 °C with shaking at 200 rpm in 100 ml of 2× TY medium (1% yeast extract, 1.5% tryptone, 0.5% NaCl) containing ampicillin (100 μg/ml) and chloramphenicol (68 μg/ml), in a 1-liter flask. Afterward, 25 ml of this culture was used to inoculate 1 liter of 2× TY medium, which was then incubated to an *A*_600 nm_ of ∼0.6 (range from 0.55 to 0.75) at 37 °C. Protein expression was induced by 0.9 mm isopropyl β-d-galactopyranoside followed by incubation for 16 h at 16 °C. The cells were harvested by centrifugation at 5000 × *g* for 15 min to yield 2–3 g/liter pellet (wet weight). The cell pellet was frozen at −80 °C overnight, thawed, and resuspended in 3 ml of 20 mm phosphate buffer, pH 7.4, 500 mm NaCl/g of wet cells. Lysonase bioprocessing reagent (Novagen, Darmstadt, Germany) was then added (4 μl/g wet cells) and incubated for 30 min on ice with rotated mixing. The cell suspension was then sonicated for a total of 1.2 min and centrifuged at 15,000 × *g* for 15 min at 4 °C; the supernatant was retained. The His_6_-tagged enzyme was purified at 4 °C after binding to a nickel-nitrilotriacetic acid His·Bind resin (Novagen, Darmstadt, Germany). The columns were pre-washed with 20 mm phosphate buffer, pH 7.4, 500 mm NaCl, and 50 mm imidazole, and the enzyme was eluted with the same buffer but containing 500 mm imidazole. The monitoring of the enzyme elution was performed by SDS-PAGE and/or activity measurements, using standard assays (see below). After elution, protein solution was extensively dialyzed with 20 mm Tris, pH 7.5, 50 mm NaCl by ultrafiltration through low adsorption hydrophilic 10,000 nominal molecular weight limit cutoff membranes (regenerated cellulose, Amicon), after which the protein was maintained at a concentration of 10 mg/ml; the protein stock solution was stored at −20 °C until used in assays. The purity was assessed as >95% using SDS-PAGE, which was performed with 12% (v/v) polyacrylamide gels, using a Bio-Rad Mini Protean system. Prior to crystallization assays, 2 mm dithiothreitol (DTT) was added.

##### Biochemical Assays

Specific activity (units/g) and kinetic parameters (*K_m_* and *k*_cat_) were first determined using *p*NP sugars (read at 405 nm) in 96-well plates, as described previously (28). *p*NP substrates tested included those containing α-glucose (*p*NPαGlc), α-maltose (*p*NPαMal), β-glucose (*p*NPβGlc), β-cellobiose (*p*NPβCel), α-arabinofuranose (*p*NPαAraf), β-arabinopyranose (*p*NPβArap), α-xylose (*p*NPαXyl), β-xylose (*p*NPβXyl), β-xylobiose (*p*NPβXylb), α-fucose (*p*NPαFuc), α-rhamnose (*p*NPαRha), α-mannose (*p*NPαMan), β-mannose (*p*NPβMan), α-galactose (*p*NPαGal), β-galactose (*p*NPβGal), β-lactose (*p*NPβLac), *N*-acetyl-β-d-glucosaminide (*p*NPGlcNAc), and *N*-acetyl-β-d-galactosaminide (*p*NPGalNAc). For cello-oligosaccharides (DP from 2 to 5), gentibiose and sophorose, the level of released glucose was determined using a glucose oxidase kit (Sigma). The level of released xylose from xylo-oligosaccharides (DP from 2 to 5) was determined using the d-xylose assay kit from Megazyme (Bray, Ireland). Substrate specificity was investigated also using carboxymethylcellulose, lichenan, barley glucan, laminarin, and avicel (all from Sigma and filter paper (Whatman, UK). Specific activity for all these sugars was quantified by measuring release of reducing sugars according to Miller (50). For *K_m_* determinations, assay reactions were conducted by adding a protein concentration of 0.23 μm to an assay mixture containing from 0 to 30 mm sugar in 50 mm sodium acetate buffer, pH 5.6, *T* = 40 °C. Total reaction volume was 200 μl. For *k*_cat_ determinations, under the same conditions, sugar concentration was set up to 2 times the *K_m_* value, and the protein concentration was from 0 to 0.23 μm. For specific activity determinations (units/g), a protein concentration of 0.23 μm and 10 mg/ml of the sugar or polysaccharide were used in 50 mm sodium acetate buffer, pH 5.6, *T* = 40 °C. The pH and temperature optima were determined in the range of pH 4.0–8.5 (50 mm Britton-Robinson buffer, BR) and 20–65 °C in assays containing a protein concentration of 0.23 μm and 10 mg/ml *p*NPβGlc, which was used as standard substrate. BR buffer is a “universal” pH buffer used for the range pH 2–12. It consists of a mixture of 0.04 m H_3_BO_3_, 0.04 m H_3_PO_4_, and 0.04 m CH_3_COOH that has been titrated to the desired pH with 0.2 m NaOH. Optimal pH was measured at 40 °C, and the optimal temperature was determined in the same buffer used in the kinetic assays. In all cases, absorbance was determined immediately after reagent and enzyme were mixed using a microplate reader every 1 min for a total time of 15 min (Synergy HT Multi-Mode Microplate Reader, BioTek). All reactions were performed in triplicate. One unit of enzyme activity was defined as the amount of enzyme required to transform 1 μmol of substrate in 1 min under the assay conditions, with extinction coefficients as in Ref. 21. All values were corrected for non-enzymatic hydrolysis (background rate). The protein concentration was determined spectrophotometrically (at 280 nm) using a BioTek EON microplate reader (Synergy HT Multi-Mode Microplate Reader, BioTek) according to extinction coefficient of the protein (108,485 m^−1^ cm^−1^) corresponding to its amino acid sequence.

Note that the detection limit, using a microplate reader with a filter for 405 nm, for the yellow chromogen is about 1·10^−6^ mol/liter *p*-nitrophenol. Because the concentration of substrate in the assay ranges from 0 to 30 mm, it is expected that detection of the activity under our assay conditions is much above the detection limit.

##### Crystallization Data Collection and Crystal Structure Determination

Initial crystallization conditions for the complete GlyA_1_ (10 mg/ml) were explored by high-throughput techniques with a NanoDrop robot (Innovadyne Technologies Inc.), using different commercial screens as follows: PACT and JCSG+ Suites from Qiagen; JBScreen Classic 1–4 from Jena Bioscience; and Index, Crystal Screen, and SaltRx packages from Hampton Research. These assays were carried out using the sitting drop vapor-diffusion method in MRC 96-well crystallization plates (Molecular Dimensions).

Elongated bars grew after 3 months in 20% polyethyleneglycol (PEG) 3350, 0.2 m ammonium sulfate, BisTris, pH 5.5. For data collection, crystals were cryoprotected in mother liquor supplemented with 25% (w/v) d-glucose before being cooled in liquid nitrogen. Diffraction data were collected at the German Electron Synchrotron (Hamburg, Germany). Diffraction images were processed with XDS (40) and scaled using Aimless from the CCP4 package (41) leading to space group P2_1_2_1_2_1_. The structure was solved by molecular replacement using MOLREP (42) with reflections up to 2.5 Å resolution range and a Patterson radius of 54 Å. The template model was the β-glucosidase from *T. neapolitana* (PDB code 2X42), but the search was made in two steps. First, the region containing residues 2–315 was used for finding a partial solution. Then, another round of molecular replacement, with the region 321–721, was computed. Preliminary rigid-body refinement was carried out using REFMAC (43). Subsequently, several rounds of extensive model building with COOT (44) combined with automatic restraint refinement with flat bulk solvent correction and using maximum likelihood target features led to a model covering residues 3–798. However, no density was found for the loop 103–108 or for the last 123 residues of the protein. At the latter stages, β-glucose, sulfate ions, and water molecules were included in the model, which, combined with more rounds of restrained refinement, led to a final *R*-factor of 15.7 (*R*_free_ 17.8). The free *R*-factor was calculated using a subset of 5% randomly selected structure-factor amplitudes that were excluded from automated refinement. Many attempts to reproduce and improve these crystals were unsuccessful, until *in situ* proteolysis of the sample with subtilisin was tried. Resulting crystals grew after 15 days in the same conditions, but at pH 7.0, they were cryoprotected in 20% (v/v) glycerol and showed the same space group and cell content. Then, the truncated GlyA_1_-ΔCt construct (residues 1–798) was tested. Initial crystallization assays were accomplished as described above, and several hits were obtained. Best crystals were grown in 2.0 m ammonium sulfate, 0.1 m BisTris, pH 5.5, and belonged to the same space group. The asymmetric unit contains a single molecule, with a Matthews's coefficient of 2.73 and a 54% solvent content within the cell.

Soaking experiments with d-xylose or d-galactose were performed with the truncated construct in mother liquor solution implemented with 5–50 mm ligand. Then, the crystals were flash-frozen into liquid nitrogen using mother liquor plus 20% (v/v) glycerol or ethylene glycol as cryoprotectants. The ligands were manually modeled into the electron density maps and were refined similarly to that described above. Although a mixture of α- and β-anomers may exist in solution, only the β-form of the monosaccharides was observed at the active site of the different complexes. For the docked glucotetraose coordinates, not present in the Protein Data Bank, a model was built by the on-line carbohydrate-building program GLYCAM (45).

Many attempts to crystallize the C-terminal section of the protein using the available construct were unsuccessful, and therefore, a model was built as explained below. The figures were generated with PyMOL (46). The atomic coordinates have been deposited in the RCSB Protein Data Bank under the accession codes 5K6I, 5K6M, 5K6N, and 5K6O.

##### SAXS Measurements

GlyA_1_ and GlyA_1_-ΔCt stock solutions (10 mg/ml) were dialyzed against the same buffer (20 mm Tris-HCl, pH 7.5, 50 mm NaCl, 2 mm DTT, and 5% glycerol) for 18 h. SAXS measurements were performed at ESRF on beamline BM29, equipped with a Pilatus 1M detector. Each sample concentration, prepared by dilution of these stock solutions, was measured in 10 frames, 1-s exposure time per frame, at 4 °C, at a sample-to-detector distance of 2.867 m, using an x-ray wavelength of 0.991 Å. No radiation damage was observed during the measurements. The SAXS curves for buffer solutions were subtracted from the protein solution curves before analysis.

The scattering curves from six gradual concentrations, from 0.3 to 5 mg/ml, were scaled and averaged to obtain the *I*(*q*) function using the ATSAS software package (47). The radius of gyration (*R_g_*) for each protein was calculated by Guinier plot using the program PRIMUS, and the pair distribution function *P*(*r*) and the maximum particle size *D*_max_ were obtained by the program GNOM. Then, POROD was used to calculate the excluded volume of the particle, as well as the molecular weight of each sample.

Several homology and threading modeling programs were tried to obtain a model of the last 123 residues of GlyA_1_. All of them predicted a topology corresponding to carbohydrate-binding domains of families CBM6/CBM35, but they differed in the length of the linker attaching this domain to the core protein. Finally, models obtained from Swiss-Model (48) and CPHmodel (49) servers were used (templates from PDB entries 2W46 and 1UYX), each predicting a loop of 32 or 5 residues, respectively. Both entries share less than 20% identity with the C-terminal region of GlyA_1_.

Subsequently, CORAL (47) was used for several rounds of two-domain rigid body fitting, using the GlyA_1_-ΔCt coordinates and both templates, alternately; linkers were built as dummy atoms. The fit of the CORAL models to the SAXS experimental data were evaluated by the χ^2^ value calculated from the program CRYSOL (47).

##### Sequence Analysis and Construction of a Neighbor-Joining Tree

The positioning of the sequence of the GlyA_1_ (α/β)_8_ barrel domain was analyzed in a phylogenetic tree. The predicted protein sequences were aligned against the National Center for Biotechnology Information non-redundant (NCBI nr) database using BLASTP algorithm. We downloaded all 27,499 GH3 sequences deposited in public databases. They were grouped within five different domain architectures as follows: ABB (9, 196), ABB_ABS (3,392), ABB_ABS_FLD (11,910), ABB_ABS_PA14_FLD (2,673), and ABS_FLD_ABB (328), where ABB, ABS, FLD, and PA14 refer to (α/β)_8_ barrel domain, (α/β)_6_-sandwich, fibronectin-like type III domain, and protective antigen PA14 domain, respectively. We discarded those sequences (848) from the ABB_ABS group longer than 700 amino acids, as they represent enzymes with unidentified domains downstream from the ABS module. Subsequently, the sequence corresponding to the ABB domain was extracted from all of the five sub-groups. An additional filter was applied to remove ABB sequences with coverage lower than 60% of the consensus domain defined by Interpro or Pfam databases (*i.e.* with less than 200 amino acids). The final number of sequences was the following: ABB (8,109), ABB_ABS (2,312), ABB_ABS_FLD (7,335), ABB_ABS_PA14_FLD (1,664), and ABS_FLD_ABB (289). For each of the five sub-groups, redundant sequences (those sharing more than 50% identity) were eliminated to select sequences that belong to different taxonomic groups. Following this procedure, the final selected sequences were as follows: ABB (132), ABB_ABS (54), ABB_ABS_FLD (45), ABB_ABS_PA14_FLD (20), and ABS_FLD_ABB (22). Multiple protein alignment was performed using ClustalW program, built into the software version 2.1. Phylogenetic analysis was conducted with the Ape package implemented for R programming language.

## Author Contributions

J. S. A., M. F., and J. P. conceived and coordinated the study. M. V. P. and M. F. contributed to screening, gene cloning, and enzyme production and characterization. P. N. G. contributed to metagenomics clone resources. J. S. A., B. G. P., and M. R. E. designed the crystallographic work and the SAXS experiments and interpreted the results. M. R. E. performed all the crystallography and SAXS experiments. J. M. N. and J. P. performed the phylogenetic analysis. J. S. A. and M. F. wrote the paper, and all authors read and commented on the manuscript.

## Footnotes

3 The abbreviations used are:

GH3: glycoside hydrolase family-3
CBM: carbohydrate-binding module
DP: degree of polymerization
*p*NP: *p*-nitrophenyl
*p*NPαGlc: *p*NP-α-glucose
*p*NPαMal: *p*NP-α-maltose
*p*NPβGlc: *p*NP-β-glucose
*p*NPβCel: *p*NP-β-cellobiose
*p*NPαAraf: *p*NP-α-arabinofuranose
*p*NPβArap: *p*NP-β-arabinopyranose
*p*NPαXyl: *p*NP-α-xylose
*p*NPβXyl: *p*NP-β-xylose
*p*NPβXylb: *p*NP-β-xylobiose
*p*NPαFuc: *p*NP-α-fucose
*p*NPαRha: *p*NP-α-rhamnose
*p*NPαMan: *p*NP-α-mannose
*p*NPβMan: *p*NP-β-mannose
*p*NPαGal: *p*NP-α-galactose
*p*NPβGal: *p*NP-β-galactose
*p*NPβLac: *p*NP-β-lactose
*p*NPGlcNAc: *p*NP-*N*-acetyl-β-d-glucosaminide
*p*NPGalNAc: *p*NP-*N*-acetyl-β-d-galactosaminide
r.m.s.d.: root mean square deviation
SRF: seminal rumen fluid
SAXS: small-angle x-ray scattering
BisTris: 2-[bis(2-hydroxyethyl)amino]-2-(hydroxymethyl)propane-1,3-diol
PDB: Protein Data Bank
FLD: fibronectin-like type III domain.

## References

[1] Cantarel B. L., Coutinho P. M., Rancurel C., Bernard T., Lombard V., and Henrissat B. (2009) The Carbohydrate-active EnZymes database (CAZy): an expert resource for glycogenomics. Nucleic Acids Res. 37, D233–D238 18838391 10.1093/nar/gkn663 PMC2686590

[2] Lee J. H., Hyun Y. J., and Kim D. H. (2011) Cloning and characterization of α-l-arabinofuranosidase and bifunctional α-l-arabinopyranosidase/β-d-galactopyranosidase from *Bifidobacterium longum* H-1. J. Appl. Microbiol. 111, 1097–1107 21851513 10.1111/j.1365-2672.2011.05128.x

[3] Mayer C., Vocadlo D. J., Mah M., Rupitz K., Stoll D., Warren R. A., and Withers S. G. (2006) Characterization of a β-*N*-acetylhexosaminidase and a β-*N*-acetylglucosaminidase/β-glucosidase from *Cellulomonas fimi*. FEBS J. 273, 2929–2941 16762038 10.1111/j.1742-4658.2006.05308.x

[4] DeBoy R. T., Mongodin E. F., Fouts D. E., Tailford L. E., Khouri H., Emerson J. B., Mohamoud Y., Watkins K., Henrissat B., Gilbert H. J., and Nelson K. E. (2008) Insights into plant cell wall degradation from the genome sequence of the soil bacterium *Cellvibrio japonicus*. J. Bacteriol. 190, 5455–5463 18556790 10.1128/JB.01701-07 PMC2493263

[5] Mai V., Wiegel J., and Lorenz W. W. (2000) Cloning, sequencing, and characterization of the bifunctional xylosidase-arabinosidase from the anaerobic thermophile *Thermoanaerobacter ethanolicus*. Gene 247, 137–143 10773453 10.1016/s0378-1119(00)00106-2

[6] Zhou J., Bao L., Chang L., Liu Z., You C., and Lu H. (2012) β-Xylosidase activity of a GH3 glucosidase/xylosidase from yak rumen metagenome promotes the enzymatic degradation of hemicellulosic xylans. Lett. Appl. Microbiol. 54, 79–87 22085266 10.1111/j.1472-765X.2011.03175.x

[7] Varghese J. N., Hrmova M., and Fincher G. B. (1999) Three-dimensional structure of a barley β-d-glucan exohydrolase, a family 3 glycosyl hydrolase. Structure 7, 179–190 10368285 10.1016/s0969-2126(99)80024-0

[8] Pozzo T., Pasten J. L., Karlsson E. N., and Logan D. T. (2010) Structural and functional analyses of β-glucosidase 3B from *Thermotoga neapolitana*: a thermostable three-domain representative of glycoside hydrolase 3. J. Mol. Biol. 397, 724–739 20138890 10.1016/j.jmb.2010.01.072

[9] Yoshida E., Hidaka M., Fushinobu S., Koyanagi T., Minami H., Tamaki H., Kitaoka M., Katayama T., and Kumagai H. (2010) Role of a PA14 domain in determining substrate specificity of a glycoside hydrolase family 3 β-glucosidase from *Kluyveromyces marxianus*. Biochem. J. 431, 39–49 20662765 10.1042/BJ20100351

[10] Nakatani Y., Cutfield S. M., Cowieson N. P., and Cutfield J. F. (2012) Structure and activity of exo-1,3/1,4-β-glucanase from marine bacterium *Pseudoalteromonas sp.* BB1 showing a novel C-terminal domain. FEBS J. 279, 464–478 22129429 10.1111/j.1742-4658.2011.08439.x

[11] Zmudka M. W., Thoden J. B., and Holden H. M. (2013) The structure of DesR from *Streptomyces venezuelae*, a β-glucosidase involved in macrolide activation. Protein Sci. 22, 883–892 23225731 10.1002/pro.2204 PMC3719083

[12] Karkehabadi S., Helmich K. E., Kaper T., Hansson H., Mikkelsen N. E., Gudmundsson M., Piens K., Fujdala M., Banerjee G., Scott-Craig J. S., Walton J. D., Phillips G. N. Jr, and Sandgren M. (2014) Biochemical characterization and crystal structures of a fungal family 3 β-glucosidase, Cel3A from *Hypocrea jecorina*. J. Biol. Chem. 289, 31624–31637 25164811 10.1074/jbc.M114.587766 PMC4223358

[13] Suzuki K., Sumitani J., Nam Y. W., Nishimaki T., Tani S., Wakagi T., Kawaguchi T., and Fushinobu S. (2013) Crystal structures of glycoside hydrolase family 3 β-glucosidase 1 from *Aspergillus aculeatus*. Biochem. J. 452, 211–221 23537284 10.1042/BJ20130054

[14] Agirre J., Ariza A., Offen W. A., Turkenburg J. P., Roberts S. M., McNicholas S., Harris P. V., McBrayer B., Dohnalek J., Cowtan K. D., Davies G. J., and Wilson K. S. (2016) Three-dimensional structures of two heavily *N*-glycosylated *Aspergillus* sp. family GH3 β-d-glucosidases. *Acta Crystallogr*. D Struct. Biol. 72, 254–265 10.1107/S2059798315024237 PMC4756609 26894673

[15] Marín-Navarro J., Gurgu L., Alamar S., and Polaina J. (2011) Structural and functional analysis of hybrid enzymes generated by domain shuffling between *Saccharomyces cerevisiae* (var. *diastaticus*) Sta1 glucoamylase and *Saccharomycopsis fibuligera* Bgl1 β-glucosidase. Appl. Microbiol. Biotechnol. 89, 121–130 20821204 10.1007/s00253-010-2845-3

[16] Litzinger S., Fischer S., Polzer P., Diederichs K., Welte W., and Mayer C. (2010) Structural and kinetic analysis of *Bacillus subtilis N*-acetylglucosaminidase reveals a unique Asp-His dyad mechanism. J. Biol. Chem. 285, 35675–35684 20826810 10.1074/jbc.M110.131037 PMC2975192

[17] Bacik J. P., Whitworth G. E., Stubbs K. A., Vocadlo D. J., and Mark B. L. (2012) Active site plasticity within the glycoside hydrolase NagZ underlies a dynamic mechanism of substrate distortion. Chem. Biol. 19, 1471–1482 23177201 10.1016/j.chembiol.2012.09.016

[18] Nakajima M., Yoshida R., Miyanaga A., Abe K., Takahashi Y., Sugimoto N., Toyoizumi H., Nakai H., Kitaoka M., and Taguchi H. (2016) Functional and structural analysis of a β-glucosidase involved in β-1,2-glucan metabolism in *Listeria innocua*. PLoS ONE 11, e0148870 26886583 10.1371/journal.pone.0148870 PMC4757417

[19] Hrmova M., De Gori R., Smith B. J., Fairweather J. K., Driguez H., Varghese J. N., and Fincher G. B. (2002) Structural basis for broad substrate specificity in higher plant β-d-glucan glucohydrolases. Plant Cell 14, 1033–1052 12034895 10.1105/tpc.010442 PMC150605

[20] Hrmova M., De Gori R., Smith B. J., Vasella A., Varghese J. N., and Fincher G. B. (2004) Three-dimensional structure of the barley β-d-glucan glucohydrolase in complex with a transition state mimic. J. Biol. Chem. 279, 4970–4980 14597633 10.1074/jbc.M307188200

[21] Hrmova M., Streltsov V. A., Smith B. J., Vasella A., Varghese J. N., and Fincher G. B. (2005) Structural rationale for low-nanomolar binding of transition state mimics to a family GH3 β-d-glucan glucohydrolase from barley. Biochemistry 44, 16529–16539 16342944 10.1021/bi0514818

[22] Yarza P., Yilmaz P., Pruesse E., Glöckner F. O., Ludwig W., Schleifer K. H., Whitman W. B., Euzéby J., Amann R., and Rosselló-Móra R. (2014) Uniting the classification of cultured and uncultured bacteria and archaea using 16S rRNA gene sequences. Nat. Rev. Microbiol. 12, 635–645 25118885 10.1038/nrmicro3330

[23] Alcaide M., Tornés J., Stogios P. J., Xu X., Gertler C., Di Leo R., Bargiela R., Lafraya A., Guazzaroni M. E., López-Cortés N., Chernikova T. N., Golyshina O. V., Nechitaylo T. Y., Plumeier I., Pieper D. H., Yakimov M. M., Savchenko A., Golyshin P. N., and Ferrer M. (2013) Single residues dictate the co-evolution of dual esterases: MCP hydrolases from the α/β hydrolase family. Biochem. J. 454, 157–166 23750508 10.1042/BJ20130552

[24] Ferrer M., Martínez-Martínez M., Bargiela R., Streit W. R., Golyshina O. V., and Golyshin P. N. (2016) Estimating the success of enzyme bioprospecting through metagenomics: current status and future trends. Microb. Biotechnol. 9, 22–34 26275154 10.1111/1751-7915.12309 PMC4720405

[25] Yoshida S., Hiraga K., Takehana T., Taniguchi I., Yamaji H., Maeda Y., Toyohara K., Miyamoto K., Kimura Y., and Oda K. (2016) A bacterium that degrades and assimilates poly(ethylene terephthalate). Science 351, 1196–1199 26965627 10.1126/science.aad6359

[26] Alcaide M., Stogios P. J., Lafraya Á., Tchigvintsev A., Flick R., Bargiela R., Chernikova T. N., Reva O. N., Hai T., Leggewie C. C., Katzke N., La Cono V., Matesanz R., Jebbar M., Jaeger K. E., et al (2015) Pressure adaptation is linked to thermal adaptation in salt-saturated marine habitats. Environ. Microbiol. 17, 332–345 25330254 10.1111/1462-2920.12660

[27] Gerlt J. A., Allen K. N., Almo S. C., Armstrong R. N., Babbitt P. C., Cronan J. E., Dunaway-Mariano D., Imker H. J., Jacobson M. P., Minor W., Poulter C. D., Raushel F. M., Sali A., Shoichet B. K., and Sweedler J. V. (2011) The enzyme function initiative. Biochemistry 50, 9950–9962 21999478 10.1021/bi201312u PMC3238057

[28] Del Pozo M. V., Fernández-Arrojo L., Gil-Martínez J., Montesinos A., Chernikova T. N., Nechitaylo T. Y., Waliszek A., Tortajada M., Rojas A., Huws S. A., Golyshina O. V., Newbold C. J., Polaina J., Ferrer M., and Golyshin P. N. (2012) Microbial β-glucosidases from cow rumen metagenome enhance the saccharification of lignocellulose in combination with commercial cellulase cocktail. Biotechnol. Biofuels 5, 73 22998985 10.1186/1754-6834-5-73 PMC3477023

[29] Ménigaud S., Mallet L., Picord G., Churlaud C., Borrel A., and Deschavanne P. (2012) GOHTAM: a website for “Genomic Origin of Horizontal Transfers, Alignment and Metagenomics”. Bioinformatics 28, 1270–1271 22426345 10.1093/bioinformatics/bts118 PMC3338014

[30] Jami E., Israel A., Kotser A., and Mizrahi I. (2013) Exploring the bovine rumen bacterial community from birth to adulthood. ISME J. 7, 1069–1079 23426008 10.1038/ismej.2013.2 PMC3660679

[31] Pitta D. W., Pinchak W. E., Indugu N., Vecchiarelli B., Sinha R., and Fulford J. D. (2016) Metagenomic analysis of the rumen microbiome of steers with wheat-induced frothy bloat. Front. Microbiol. 7, 689 27242715 10.3389/fmicb.2016.00689 PMC4863135

[32] McAndrew R. P., Park J. I., Heins R. A., Reindl W., Friedland G. D., D'haeseleer P., Northen T., Sale K. L., Simmons B. A., and Adams P. D. (2013) From soil to structure, a novel dimeric β-glucosidase belonging to glycoside hydrolase family 3 isolated from compost using metagenomic analysis. J. Biol. Chem. 288, 14985–14992 23580647 10.1074/jbc.M113.458356 PMC3663519

[33] Holm L., and Rosenström P. (2010) Dali server: conservation mapping in 3D. Nucleic Acids Res. 38, W545–W549 20457744 10.1093/nar/gkq366 PMC2896194

[34] Hrmova M., Varghese J. N., De Gori R., Smith B. J., Driguez H., and Fincher G. B. (2001) Catalytic mechanisms and reaction intermediates along the hydrolytic pathway of a plant β-d-glucan glucohydrolase. Structure 9, 1005–1016 11709165 10.1016/s0969-2126(01)00673-6

[35] Hegyi H., and Gerstein M. (2001) Annotation transfer for genomics: measuring functional divergence in multi-domain proteins. Genome Res. 11, 1632–1640 11591640 10.1101/gr.183801 PMC311165

[36] Bashton M., and Chothia C. (2002) The geometry of domain combination in proteins. J. Mol. Biol. 315, 927–939 11812158 10.1006/jmbi.2001.5288

[37] Ricard G., McEwan N. R., Dutilh B. E., Jouany J. P., Macheboeuf D., Mitsumori M., McIntosh F. M., Michalowski T., Nagamine T., Nelson N., Newbold C. J., Nsabimana E., Takenaka A., Thomas N. A., Ushida K., et al (2006) Horizontal gene transfer from bacteria to rumen ciliates indicates adaptation to their anaerobic, carbohydrates-rich environment. BMC Genomics 7, 22 16472398 10.1186/1471-2164-7-22 PMC1413528

[38] Berg Miller M. E., Yeoman C. J., Chia N., Tringe S. G., Angly F. E., Edwards R. A., Flint H. J., Lamed R., Bayer E. A., and White B. A. (2012) Phage-bacteria relationships and CRISPR elements revealed by a metagenomic survey of the rumen microbiome. Environ. Microbiol. 14, 207–227 22004549 10.1111/j.1462-2920.2011.02593.x

[39] Hemsley A., Arnheim N., Toney M. D., Cortopassi G., and Galas D. J. (1989) A simple method for site-directed mutagenesis using the polymerase chain reaction. Nucleic Acids Res. 17, 6545–6551 2674899 10.1093/nar/17.16.6545 PMC318348

[40] Kabsch W. (2010) XDS. Acta Crystallogr. D Biol. Crystallogr. 66, 125–132 20124692 10.1107/S0907444909047337 PMC2815665

[41] Winn M. D., Ballard C. C., Cowtan K. D., Dodson E. J., Emsley P., Evans P. R., Keegan R. M., Krissinel E. B., Leslie A. G., McCoy A., McNicholas S. J., Murshudov G. N., Pannu N. S., Potterton E. A., Powell H. R., et al (2011) Overview of the CCP4 suite and current developments. Acta Crystallogr. D Biol. Crystallogr. 67, 235–242 21460441 10.1107/S0907444910045749 PMC3069738

[42] Vagin A., and Teplyakov A. (2010) Molecular replacement with MOLREP. Acta Crystallogr. D Biol. Crystallogr. 66, 22–25 20057045 10.1107/S0907444909042589

[43] Murshudov G. N., Vagin A. A., and Dodson E. J. (1997) Refinement of macromolecular structures by the maximum-likelihood method. Acta Crystallogr. D Biol. Crystallogr. 53, 240–255 15299926 10.1107/S0907444996012255

[44] Emsley P., and Cowtan K. (2004) Coot: model-building tools for molecular graphics. Acta Crystallogr. D Biol. Crystallogr. 60, 2126–2132 15572765 10.1107/S0907444904019158

[45] Kirschner K. N., Yongye A. B., Tschampel S. M., González-Outeiriño J., Daniels C. R., Foley B. L., and Woods R. J. (2008) GLYCAM06: a generalizable biomolecular force field. Carbohydrates. J. Comput. Chem. 29, 622–655 17849372 10.1002/jcc.20820 PMC4423547

[46] DeLano W. L. (2002) The PyMOL Molecular Graphics System, version 1.6, DeLano Scientific, San Carlos, CA

[47] Petoukhov M. V., Franke D., Shkumatov A. V., Tria G., Kikhney A. G., Gajda M., Gorba C., Mertens H. D., Konarev P. V., and Svergun D. I. (2012) New developments in the program package for small-angle scattering data analysis. J. Appl. Crystallogr. 45, 342–350 25484842 10.1107/S0021889812007662 PMC4233345

[48] Biasini M., Bienert S., Waterhouse A., Arnold K., Studer G., Schmidt T., Kiefer F., Gallo Cassarino T., Bertoni M., Bordoli L., and Schwede T. (2014) SWISS-MODEL: modelling protein tertiary and quaternary structure using evolutionary information. Nucleic Acids Res. 42, W252–W258 24782522 10.1093/nar/gku340 PMC4086089

[49] Nielsen M., Lundegaard C., Lund O., and Petersen T. N. (2010) CPHmodels-3.0–remote homology modeling using structure-guided sequence profiles. Nucleic Acids Res. 38, W576–W581 20542909 10.1093/nar/gkq535 PMC2896139

[50] Miller G. L. (1959) Use of dinitrosalicylic acid reagent for determination of reducing sugar. Anal. Chem. 31, 426–428

